# MYPT1 inhibits the metastasis of renal clear cell carcinoma via the MAPK8/N‐cadherin pathway

**DOI:** 10.1002/2211-5463.13487

**Published:** 2022-09-27

**Authors:** Qingling Xie, Ren Liu, Zhihao Zou, Yuanfa Feng, Yiqiao Huang, Guibin Xu, Wei Sun, Yuxiang Liang, Weide Zhong

**Affiliations:** ^1^ Guangdong Provincial Institute of Nephrology, Nanfang Hospital Southern Medical University Guangzhou China; ^2^ Department of Urology, Guangdong Key Laboratory of Clinical Molecular Medicine and Diagnostics, Guangzhou First People's Hospital, School of Medicine South China University of Technology Guangzhou China; ^3^ Department of Urology The Fifth Affiliated Hospital of Guangzhou Medical University China; ^4^ Department of Urology, Huadu District People's Hospital Southern Medical University Guangzhou China

**Keywords:** cadherin, MAPK, metastasis, MYPT1, renal clear cell carcinoma

## Abstract

Myosin phosphatase target subunit 1 (MYPT1) is a subunit of myosin phosphatase that is capable of regulating smooth muscle contraction. MYPT1 has been reported to be involved in a wide variety of tumours, but its expression and biological functions in renal clear cell carcinoma (ccRCC) remain obscure. Herein, we analysed the relationship between patient clinicopathological characteristics and MYPT1 expression levels in ccRCC patients using a tissue microarray (TMA) and data retrieved from the TCGA‐KIRC dataset. MYPT1 was overexpressed or depleted using siRNA in ccRCC cells to assess the effects on migration and invasion *in vitro* and *in vivo*. Additionally, RNA‐sequencing and bioinformatics analysis were performed to investigate the precise mechanism. MYPT1 expression in ccRCC tissues was observed to be lower than that in nonmalignant tissues (*P* < 0.05). In addition, MYPT1 downregulation was closely linked to advanced pathological stage (*P* < 0.05), and poor OS (overall survival; *P* < 0.05). Functionally, increased expression of MYPT1 suppressed ccRCC migration and invasion *in vitro*, and inhibited tumour metastasis *in vivo*. In addition, MYPT1 overexpression exerted its suppressive effects via the MAPK8/N‐cadherin pathway in ccRCC.

## Graphical abstract

In this study, a MYPT1‐MAPK8/N‐cadherin axis in tumour cells was revealed to inhibit the metastasis of renal clear cell carcinoma. MYPT1 expression was downregulated in tumour tissues and linked to aggressive disease progression and a poor prognosis in ccRCC patients.

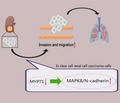

**Abbreviations**
ccRCC: renal clear cell carcinoma
EMT: epithelial‐mesenchymal transition
GSEA: Gene Set Enrichment Analysis
IHC: immunohistochemistry
KEGG: Kyoto Encyclopedia of Genes and Genomes
MAPK8: mitogen‐activated protein kinase
MYPT1: myosin phosphatase target subunit 1
OS: overall survival
TCGA: The Cancer Genome Atlas
TMA: tissue microarray

Renal cell carcinoma (RCC) is among the most common lethal urologic cancers, and its incidence has increased recently. Clear cell renal cell carcinoma (ccRCC) represents the most prevalent type of renal carcinoma (~ 75%). Almost one‐third of all individuals with ccRCC have metastatic dissemination at presentation and nearly half of all patients die from their disease [1]. Surgery is the current major therapeutic approach for ccRCC patients, but approximately 30–40% of individuals with localized ccRCC develop metastatic relapse in the course of follow‐up after surgical resection and are unresponsive to chemotherapy and radiotherapy [2]. At present, the treatment of metastatic RCC is mainly based on targeted therapy, including vascular endothelial growth factor receptor inhibitors, mTOR pathway inhibitors and immune checkpoint inhibitors. However, the efficacy of many available drugs is limited due to low efficacy, high toxicity, and high drug resistance potential. Therefore, it is necessary and urgent to find new targets and possible molecular pathways to exploit for ccRCC treatment.

Myosin phosphatase target subunit 1 (MYPT1) is one of the subunits of myosin phosphatase; it functions as a modulatory subunits that regulate the subcellular localization and specificity of the respective substrates [3]. The MYPT family members possess numerous common conserved domains, including the RVxF motif for PP1c docking and many ankyrin repeats, which functionally participate in various pathological events, such as hypertension, diseases of the nervous system, and cancers [4]. MYPT1 plays an indispensable role in modulating smooth muscle contraction [5, 6]; however, other roles of MYPT1 have been recently documented; for instance, it is involved in migration, cell adhesion [7], and the cell cycle [8, 9] coupled with development [10]. Recently, Munoz‐Galvan et al. [11] reported that downregulation of MYPT1 enhances tumour resistance in ovarian cancer by targeting the Hippo cascade and promoting stemness. A previous investigation established that MYPT1 functions as a direct target of microRNA‐30d [12], suppresses angiogenesis, and is related to an improved prognosis in individuals with PCa, illustrating that MYPT1 could be a possible candidate drug for treating cancer [13]. Nonetheless, the clinical importance of MYPT1 and its role in ccRCC are unclear.

To address this problem, we explored the relationship of MYPT1 content with the clinicopathological properties in individuals with ccRCC in a TMA dataset and a public cancer database (The Cancer Genome Atlas). Cells with MYPT1 overexpression and knockdown were developed to assess the effects of MYPT1 on migration and infiltration *in vitro* and *in vivo*. Mechanistically, through transcriptome sequencing, we illustrated that MYPT1 plays an indispensable role in ccRCC metastasis by suppressing the MAPK8/N‐cadherin cascade, which might be important for developing novel approaches for treating individuals with ccRCC with metastasis.

## Materials and methods

### Tissue microarray (TMA) and cell lines

The ccRCC TMA (#HKidE180Su02, Shanghai Outdo Biotech Co. Ltd, Shanghai, China), which included 150 ccRCC tissues and 30 normal kidney tissues, was employed to perform immunohistochemistry assays. Corresponding clinical data included patient age, sex, clinical stage, pathology grade and overall survival (OS). We defined OS as the period from the surgery date to the last follow‐up or death. No patient was treated with chemotherapy or radiotherapy prior to the surgery. The human kidney cancer cell lines Caki1 and 769P were commercially provided by American Type Culture Collection (ATCC) and cultured as per ATCC's guidelines. The patients provided written informed consent for the use of their tissue samples. This study has been approved by the Human Ethics Committee of the Public Health Department of the People's Republic of China. The protocols conformed to the guidelines set by the Declaration of Helsinki.

### Plasmid, cell transfection and RNA interference

Lentiviruses for MYPT1 as well as the negative control were packaged with psPAX2, pMD.2G and an encoding vesicular stomatitis virus into HEK293T cells. To develop stable cell lines, the created lentivirus was directly introduced into Caki1 cells and inoculated at 37 °C for 72 h before flow cytometric analysis. Human MYPT1‐specific siRNA, human MAPK8‐specific siRNA and negative control siRNA were provided by GenePharma (Suzhou, China). The target sequences of the siRNAs were as follows: si‐MYPT1#1: 5′‐GCAGCUGCUAAAGGCUAUATT‐3′; si‐MYPT1#2: 5′‐GCAGGCUAUGAUGUUAAUATT‐3′; si‐MAPK8#1: 5'‐GCCGACCAUUUCAGAAUCATT‐3′; si‐MAPK8#2: 5'‐GCUGGUAAUAGAUGCAUCUTT‐3′; si‐negative control: 5'‐UUCUCCGAACGUACGUTT‐3′.

### Immunohistochemistry

MYPT1 protein expression in the ccRCC tissue microarray was assessed via immunohistochemistry (IHC) as documented previously [14]. The MYPT1 antibody was acquired from ABclonal (A6700, Wuhan, China).

### Western blotting

Western blotting was performed as per the protocol of our previous investigations [14]. Antibodies specific for MYPT1 (22117‐1‐AP), MAPK8 (66210‐1‐Ig), N‐cadherin (22018‐1‐AP), E‐cadherin (20874‐1‐AP) and Vimentin (10366‐1‐AP) were purchased from Proteintech Group, Inc (Chicago, IL, USA). Antibodies specific for phospho‐MYPT1 (Thr696 and Thr 853), PPP1CA and PPP1CB were purchased from Biogot Technology Co., Ltd. (Nanjing, China).

### Cell migration and invasion assays

We inoculated the ccRCC cells into six‐well plates and allowed them to grow to 90% confluence. Thereafter, the cells were inoculated with FBS for 24 h, and then scratches were made on every well with a 1000 μL sterile pipette tip. After every 12 h, images were acquired to assess the healing process via a microscope camera. After that, the healed area was computed with imagej (National Institutes of Health, Bethesda, MD, USA). In the invasion assay, 100 μL per well Matrigel (BD Biosciences, SanDiego, CA, USA) was inoculated into the upper compartment and then placed onto the 24‐well plates (Corning Life Sciences, Tewksbury, MA, USA). Next, we inoculated 3 × 10^4^ cells dispersed in 100 μL serum‐free medium into the upper compartment. In the lower compartment, we introduced 500 μL normal medium enriched with 10% FBS. After 24 h, we utilized a cotton swab to remove the upper compartment cells. Afterwards, we fixed the cells with 4% PFA and stained them (in 0.1% crystal violet) at room temperature for 30 min. Thereafter, the images of infiltrating cells were taken under a microscopy, and the cells were counted in five random fields. The experiment was replicated three times.

### *In vivo* mouse experiments

All animal experiments were performed according to the guidelines of the Institute for Laboratory Animal Research of Guangzhou Medical University (Guangzhou, China). For tail‐vein injection lung metastasis assays, 16 four‐week‐old BALB/c nude mice were commercially acquired from the Experimental Animal Center of Sun Yat‐sen University (Guangzhou, China). Caki1‐NC and Caki1‐MYPT1 cells (1 × 10^6^) were intravenously inoculated into every mouse via the tail vein. To assess tumour growth along with metastasis, the experimental mice were analysed by IVIS (*In Vivo* Imaging System) Spectrum. After 7 weeks, the mice were sacrificed and we removed the lungs of every mouse and then counted the pulmonary metastatic nodules. This study was approved by the Ethics Committee of Guangzhou First People's Hospital (approval No. 82072813).

### RNA‐Seq and bioinformatic analysis

RNA‐sequencing of Caki1‐MYPT1 and Caki1‐NC cells was performed at Gene *Denovo* (Guangzhou, China). Collection, processing, and library preparation of mRNA samples were performed as documented by the manufacturer kits. Differential expression analysis of Caki1‐MYPT1 and Caki1‐NC cells was performed with the DESeq2 r package, with ¦log2(fold change)¦ > 1 along with *P* < 0.05. We employed Metascape (https://metascape.org/gp/index.html) to perform KEGG functional enrichment along with GSEA.

The relationship of mRNA contents with *MYPT1* contents was assessed using data for 539 patient ccRCC tissues and 72 paired kidney tissues obtained from The Cancer Genome Atlas (TCGA) database (https://portal.gdc.cancer.gov).

### Statistical analysis

Statistical analyses were performed in spss v24.0 software (IBM SPSS, Chicago, IL,USA) along with graphpad prism 7 (https://www.graphpad.com/). Comparisons were performed via paired Student's *t* test or variance analysis (ANOVA). We employed Kaplan–Meier survival curves to determine survival time and log‐rank tests to perform comparisons. Cox regression was performed for univariate and multivariate analyses. *P* < 0.05 signified statistical significance.

## Results

### MYPT1 expression is downregulated in ccRCC tissues

**Fig. 1:**
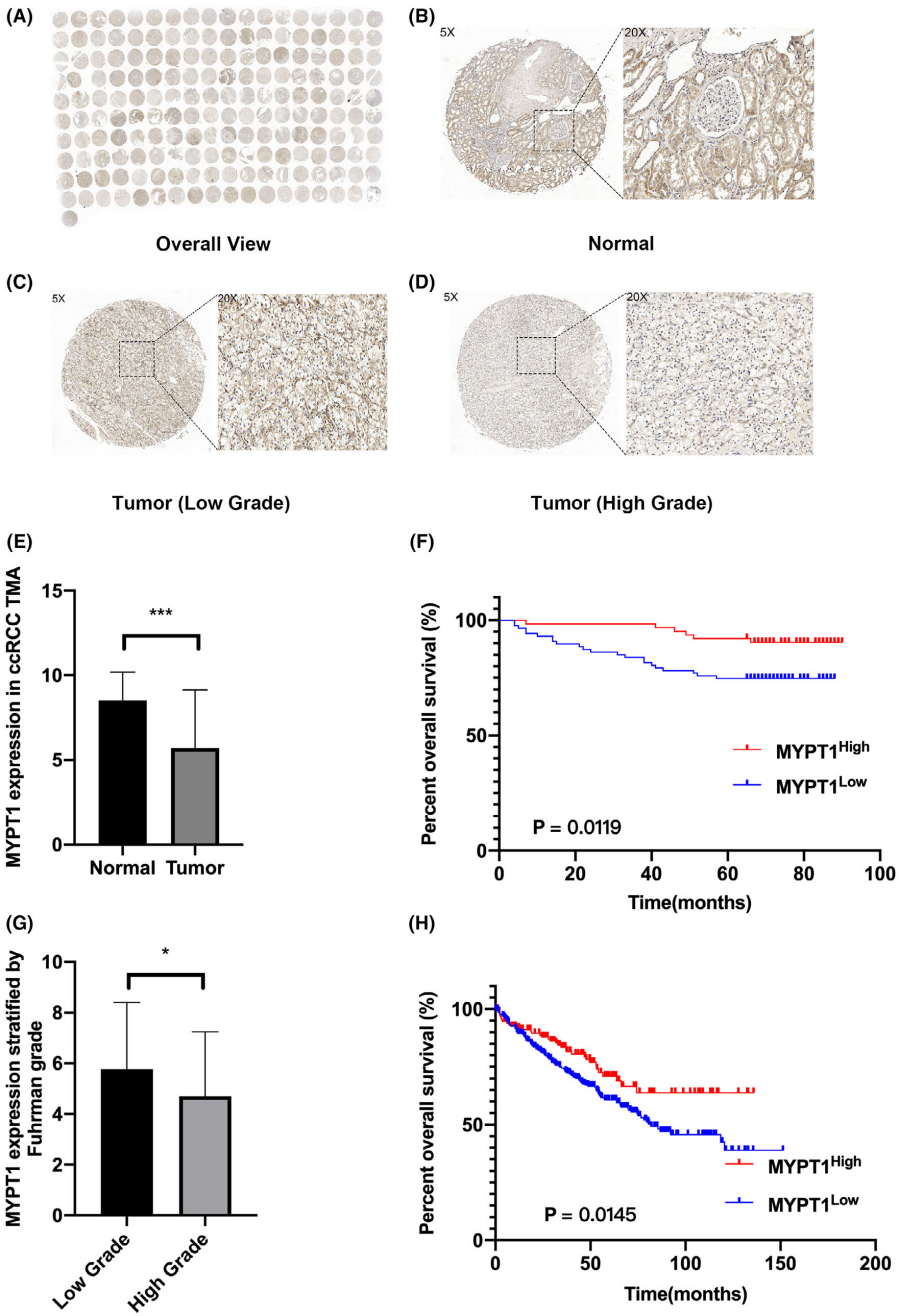
The expression of MYPT1 is downregulated in ccRCC tissues and linked to aggressive disease progression and a poor prognosis in ccRCC patients. (A) Overall view of MYPT1 immunostaining in 180 kidney samples of the TMA (150 ccRCC tissues and 30 normal kidney tissues). (B–D) Immunostaining of MYPT1 protein in normal kidney tissues, low Fuhrman grade (I/II) tumour tissues and high Fuhrman grade (III/IV) tumour tissues. (E) The histogram indicates the IHC scores of MYPT1 expression in normal and tumour tissues. (F) Kaplan–Meier curve for OS between the high MYPT1 expression group and the low MYPT expression group according to MYPT1 IHC scores (8 ~ 12, high MYPT1 expression, *n* = 55 vs. 0 ~ 6, low MYPT1 expression, *n* = 95). (G) The histogram indicates the IHC scores of MYPT1 expression in two groups stratified by Furman grade. (H) Kaplan–Meier curve for OS between the MYPT1 low and high expression groups of TCGA‐KIRC patients stratified by the median of MYPT1 expression level. Data presented as mean ± SEM (*n* = 3). Statistical analysis was performed with a two‐tailed unpaired Student's *t* test (NS, *P* > 0.05, *P < 0.05, **P < 0.01, ***P < 0.001). ccRCC, renal clear cell carcinoma; IHC, immunohistochemistry; MYPT1, myosin phosphatase target subunit 1; TMA, tissue microarray.

The expression levels of MYPT1 protein in ccRCC and normal kidney tissues were examined via immunohistochemistry (IHC) in a TMA including 150 ccRCC samples and 30 paracancer samples. As indicated in Fig. 1A–D, MYPT1 expression was primarily observed in the cytoplasm and nucleus of stained cells in tubules, and IHC analysis showed that 100% (30/30) of the normal tissues exhibited high MYPT1 expression (MYPT1++ or MYPT1+++) and 76% (114/150) of the tumour tissues exhibited low MYPT1 expression (MYPT1− or MYPT1+). MYPT1 expression was markedly reduced in ccRCC tissues compared with nonmalignant tissues (IRS: normal = 8.533 ± 1.655 vs. ccRCC = 5.433 ± 2.646, *P* < 0.001; Fig. 1E). Moreover, high protein expression of MYPT1 in ccRCC tissues was closely linked with low Furman grade (IRS: grade (I/II) = 5.767.38 ± 2.635 vs. grade (I/IV) = 4.702 ± 2.545, *P* = 0.0217; Fig. 1G).

### Downregulation of MYPT1 is linked to aggressive disease progression and a poor prognosis in ccRCC patients

As shown in Table 1, to assess the role of MYPT1 in ccRCC, the correlations between MYPT1 expression levels and the clinicopathological parameters of ccRCC patients from our TMA dataset and the TCGA‐KIRC dataset were analysed. The expression level of MYPT1 in ccRCC tissues was closely linked with low Fuhrman grade (for IHC data: *P* = 0.0217, for TCGA‐KIRC data: *P* = 0.0005, Table 1) and vital status (alive; for IHC data: *P* < 0.001, Table 1); however, there were no significant associations between MYPT1 expression and tumour stage or tumour metastasis. Using the tissues and the follow‐up information from patients, survival analyses were performed. Kaplan–Meier survival curves indicated that high MYPT1 protein expression was closely linked to better OS (log‐rank test, *P* = 0.0119; Fig. 1F). High MYPT1 mRNA expression also indicated better OS in the TCGA‐KIRC cohort (Fig. 1H).

**Table 1:** Association of MYPT1 with clinicopathological characteristics of ccRCC in TMA and TCGA‐KIRC dataset.

Clinical features	MYPT1 expression in TMA			MYPT1 expression in TCGA‐KIRC dataset		
	*n*	Means ± SD	*P*	*n*	Means ± SD	*P*
Age						
<65 years	120	5.533 ± 2.609	0.3562	332	2.661 ± 0.4428	0.0266*
≥65 years	30	5.033 ± 2.798		194	2.572 ± 0.4408	
Sex						
Female	43	5.930 ± 3.058	0.1454	183	2.629 ± 0.4168	0.9521
Male	107	5.234 ± 2.448		343	2.627 ± 0.4581	
Fuhrman						
1/2	103	5.767 ± 2.635	0.0217*	239	2.704 ± 0.4660	0.0005*
3/4	47	4.702 ± 2.545		279	2.570 ± 0.4073	
Tumour stage						
I/II	138	5.543 ± 2.609	0.0838	318	2.648 ± 0.4649	0.1852
III/IV	12	4.167 ± 2.855		206	2.595 ± 0.4100	
Metastasis						
M0				418	2.644 ± 0.4507	0.2353
M1				78	2.579 ± 0.3947	
Vital status						
Alive	122	5.820 ± 2.597	0.0001*	355	2.651 ± 0.4613	0.0840
Dead	28	3.750 ± 2.188		171	2.580 ± 0.4019	

* *P*‐value < 0.05.

### MYPT1 overexpression or knockdown influenced the migration and invasion of ccRCC cells

**Fig. 2:**
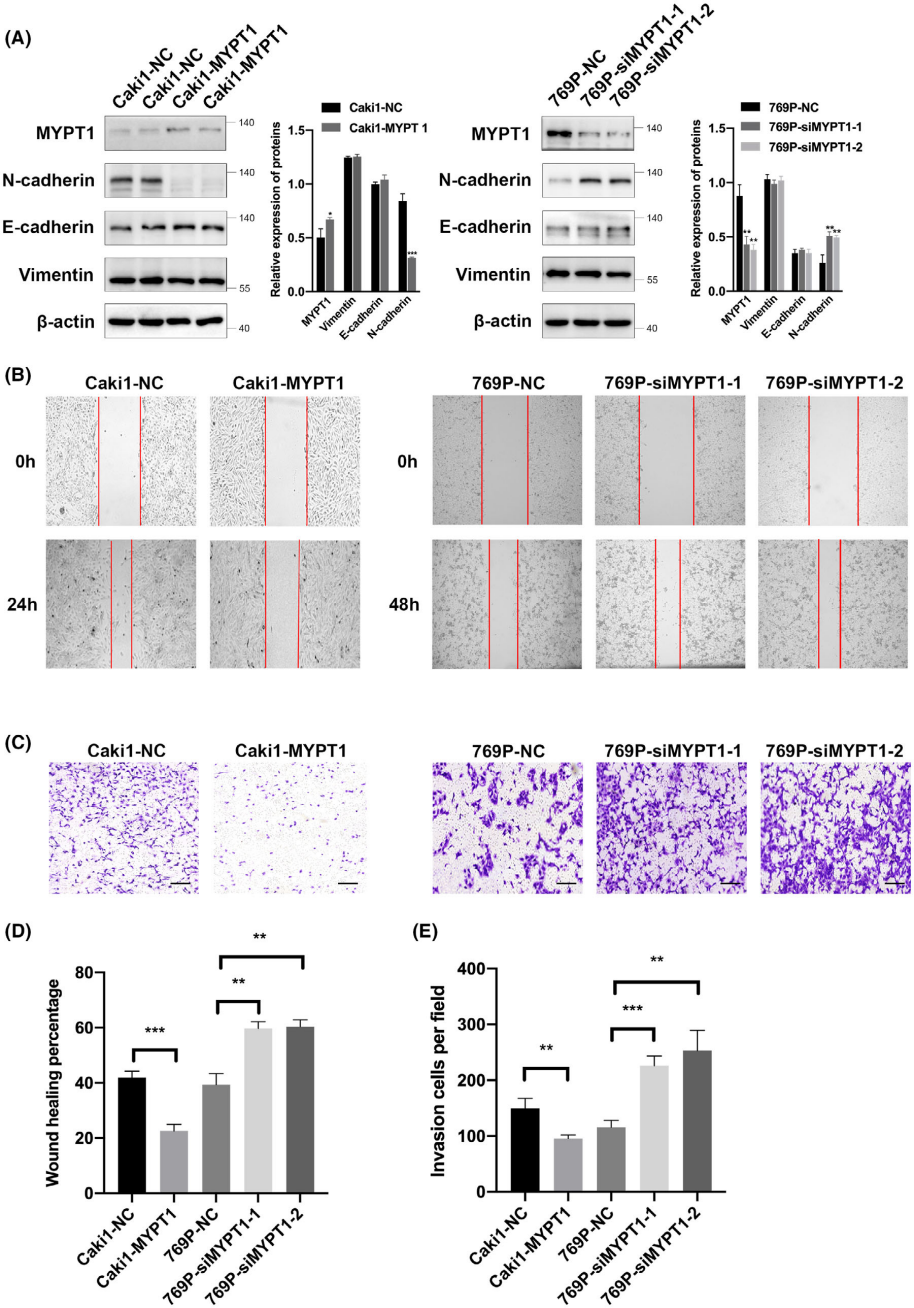
MYPT1 overexpression or knockdown influenced the migration and invasion of ccRCC cells. (A) Proteins were analysed by western blotting using the indicated antibodies. The bar plots show the relative band intensities (mean ± SEM) of three independent experiments. (B–E) Wound healing and Transwell assays were performed to determine the cell migration and invasion abilities. Red lines denote the margins of the wound. Matrigel was added to the upper chamber. Scale bars: 200 μm. Statistical analysis was performed with a two‐tailed unpaired Student's *t* test (*P < 0.05, **P < 0.01, ***P < 0.001). ccRCC, renal clear cell carcinoma; MYPT1, myosin phosphatase target subunit 1.

To further explore the biological function of MYPT1 in ccRCC, two different expression patterns of MYPT1 on the basis of its relative expression levels in ccRCC cell lines were constructed (Fig. S1A). MYPT1 or empty vector plasmid was stably transfected into the Caki1 cell line (Caki1‐MYPT1 or Caki1‐NC). Conversely, in the 769P cell line, we established MYPT1 knockdown cells (769P‐siMYPT1‐1, 769P‐siMYPT1‐2) and negative control cells (769P‐NC). Western blotting was employed to assess the expression levels of MYPT1 in MYPT1‐overexpressing or MYPT1‐silenced cell lines (Fig. 2A). Functional assays were performed to investigate the tumourigenic potential of MYPT1. The data illustrated that the overexpression or knockdown of MYPT1 did not influence ccRCC cell proliferation (Fig. S1B). Cancer cell infiltration and migration are remarkable events in ccRCC metastasis. Thus, we explored the effects of MYPT1 on ccRCC cell infiltration and migration. Fig. 2B–E showed that the overexpression of MYPT1 in ccRCC cells suppressed cell migration and invasion ability, whereas silencing the expression of MYPT1 promoted cell migration and invasion. Epithelial‐mesenchymal transition (EMT) cascades have been documented to participate in cancer cell infiltration and migration; thus, we assessed the effects of MYPT1 on EMT by exploring the expression of N‐cadherin, E‐cadherin and Vimentin. Western blotting further demonstrated that the N‐cadherin level was reduced in MYPT1‐overexpressing ccRCC cells. In contrast, MYPT1 knockdown in ccRCC cells was linked to increased N‐cadherin protein levels (Fig. 2A).

### MYPT1 suppressed ccRCC metastasis *in vivo*

**Fig. 3:**
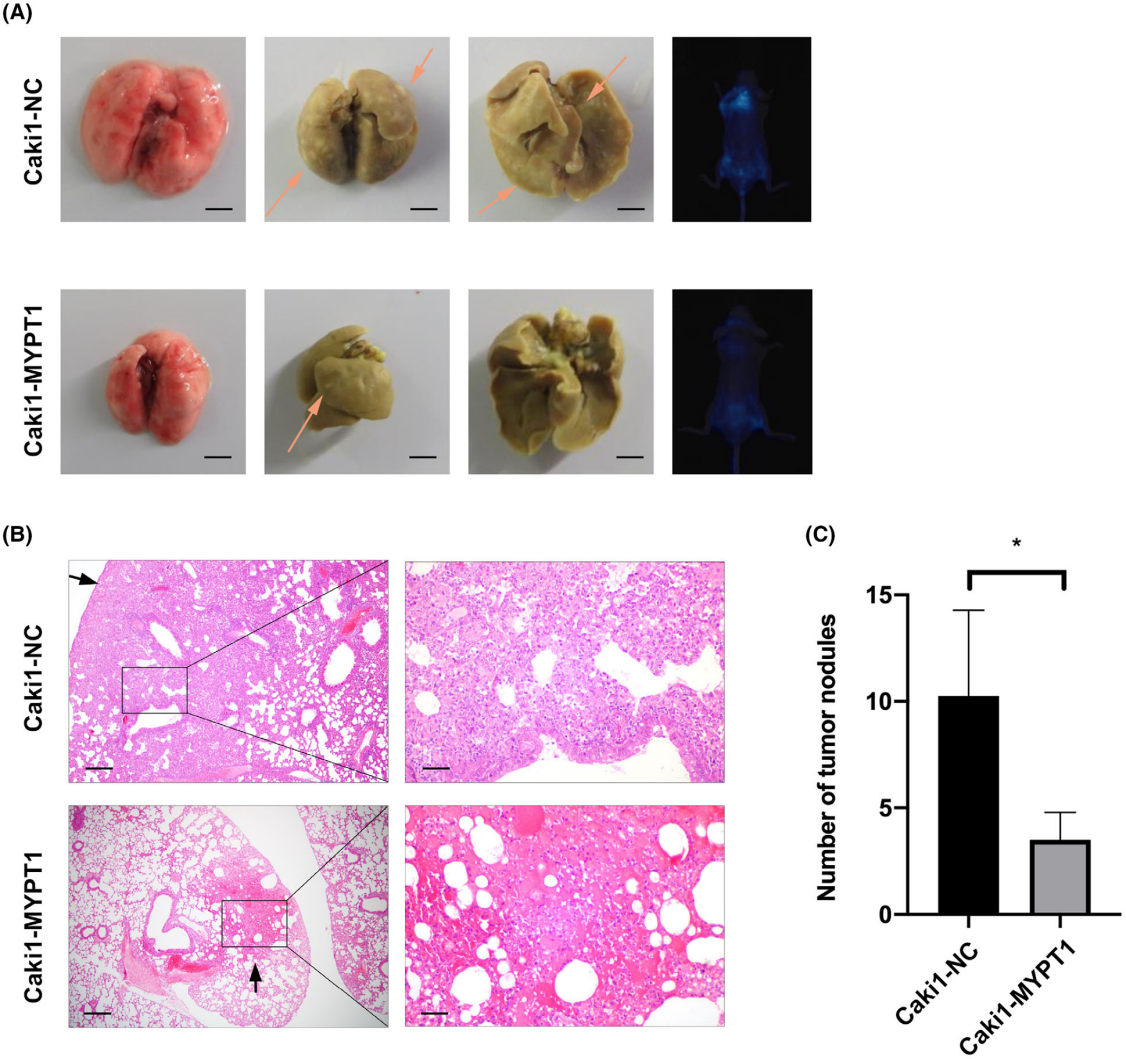
MYPT1 suppressed ccRCC metastasis *in vivo*. (A) The lung metastasis model showed that MYPT1 overexpression significantly inhibited ccRCC lung metastasis. Representative gross images of the lungs and bioluminescence images of lung metastases are shown. Arrows indicate pulmonary metastatic nodules. Scale bars: 500 μm. (B, C) Nude mice injected with MYPT1‐overexpressing Caki1 cells had fewer and smaller lung metastases. Representative images of HE staining of the lungs and statistical analysis results are shown. Scale bars: 200 or 50 μm. The pulmonary metastatic nodules were counted under a microscope, and the results were summarized. Data presented as mean ± SEM (*n* = 5). Statistical analysis was performed with a two‐tailed unpaired Student's *t* test (*P < 0.05). ccRCC, renal clear cell carcinoma; HE, haematoxylin–eosin; MYPT1, myosin phosphatase target subunit 1.

To assess the potential role of MYPT1 *in vivo*, a tail‐vein injection mouse model was utilized, and the data showed that MYPT1 overexpression markedly attenuated the lung metastatic ability of Caki1 cells (Fig. 3A–C). Altogether, our data illustrate that MYPT1 may remarkably inhibit ccRCC metastasis *in vitro* and *in vivo*.

### RNA sequencing and bioinformatic analyses demonstrated that MYPT1 is involved in a MAPK‐related cascade in ccRCC

**Fig. 4:**
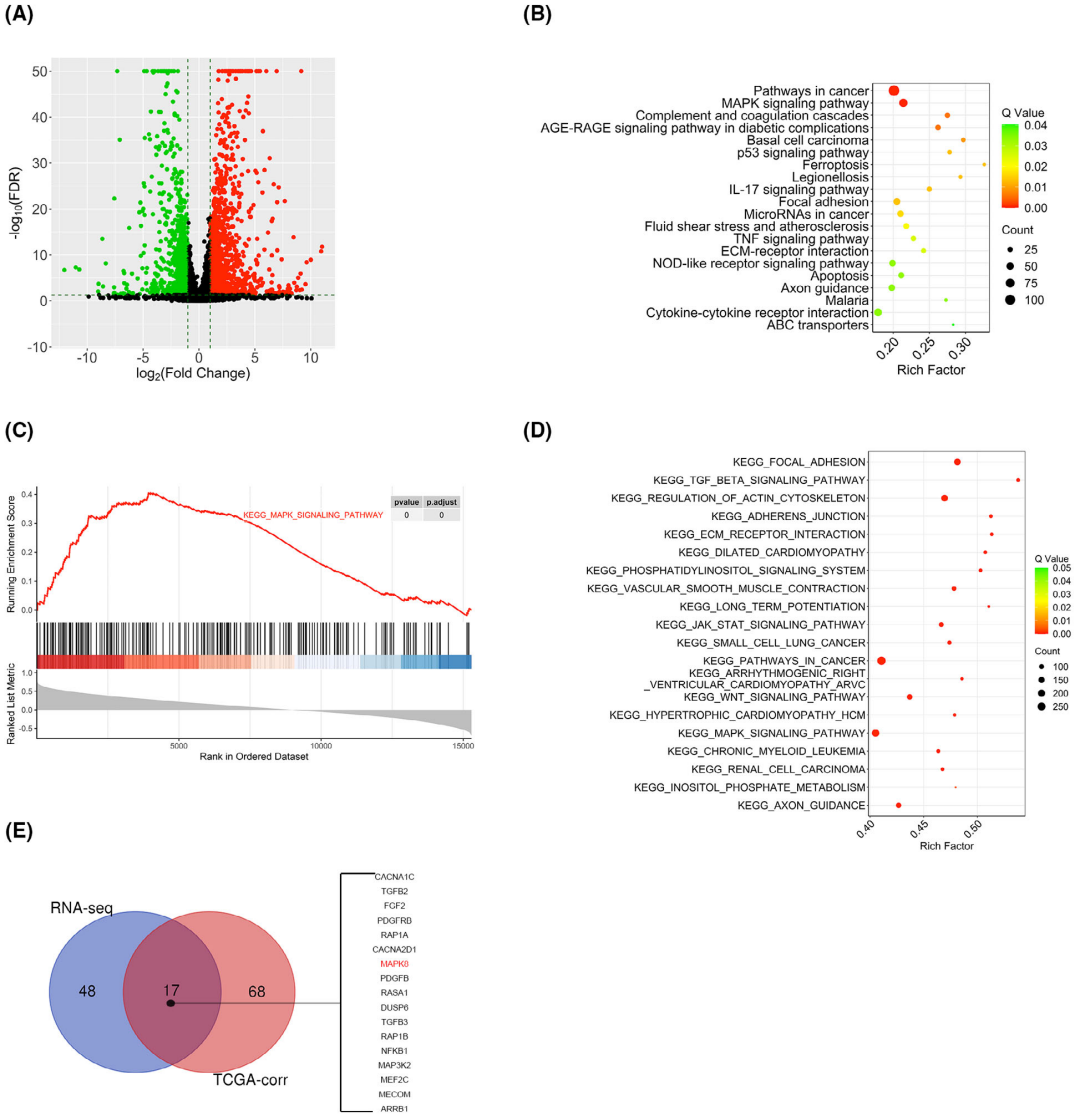
RNA sequencing and bioinformatic analyses demonstrated that MYPT1 is involved in a MAPK‐related pathway in ccRCC. (A) The volcano plot shows genes differentially expressed between Caki1 cells with MYPT1 overexpression group and the negative control group (*P* < 0.05). (B) KEGG analysis of the differentially expressed genes (¦log2FC¦ > 1, FDR‐adjusted *P* < 0.05) was performed in Caki1‐NC and Caki1‐MYPT1 cells. (C) GSEA revealed that the MAPK signalling pathway gene set was less enriched in Caki1‐MYPT1 cells (FDR < 25% and *P* < 0.05). (D) KEGG analysis of the MYPT1‐correlated genes (*P* < 0.05) in the TCGA‐KIRC dataset. (E) The 17 overlapping genes were enriched in the MAPK pathway based on the differentially expressed genes from RNA sequencing data or MYPT1‐correlated genes from TCGA‐KIRC dataset. ccRCC, renal clear cell carcinoma; GSEA, Gene Set Enrichment Analysis; KEGG, Kyoto Encyclopedia of Genes and Genomes; MAPK, mitogen‐activated protein kinase; MYPT1, myosin phosphatase target subunit 1.

Recent investigations have documented that MYPT1 plays diverse biological roles in various tumours or diseases; nonetheless, its biological role in ccRCC remains unclear. RNA sequencing analysis was performed to explore the molecular basis of the role of MYPT1 in ccRCC cells. The volcano plot of Caki1 cells exhibited the mRNAs with differential levels between the MYPT1 overexpression group and the negative controls (Fig. 4A). Based on the RNA‐seq data, Kyoto Encyclopedia of Genes and Genomes (KEGG) functional enrichment assessments and Gene Set Enrichment Analysis (GSEA) were conducted. The results suggested that the MYPT1 mainly exerts its function via the MAPK signalling cascade (Fig. 4B), and the gene sets that were positively linked to the MAPK cascade were less enriched in the group overexpressing MYPT1 (FDR < 25% and *P* < 0.05; Fig. 4C). To gain insight into the molecular mechanism linked to MYPT1 expression levels, we searched for genes whose expression was linked to that of MYPT1 in tumour samples from the TCGA‐KIRC data resources. KEGG functional enrichment analysis of these genes revealed a variety of enriched biological processes, including the MAPK signalling pathway (Fig. 4D). Hence, these data suggest a role for MAPK in MYPT1's biological function in ccRCC cells. We cross‐analysed the differentially expressed genes and MYPT1‐correlated genes enriched in the MAPK cascade and found that 17 genes may be involved in the metastasis suppression effect of MYPT1 (Fig. 4E).

### MYPT1 suppressed MAPK8/N‐cadherin expression to influence the migration and invasion of ccRCC cells

**Fig. 5:**
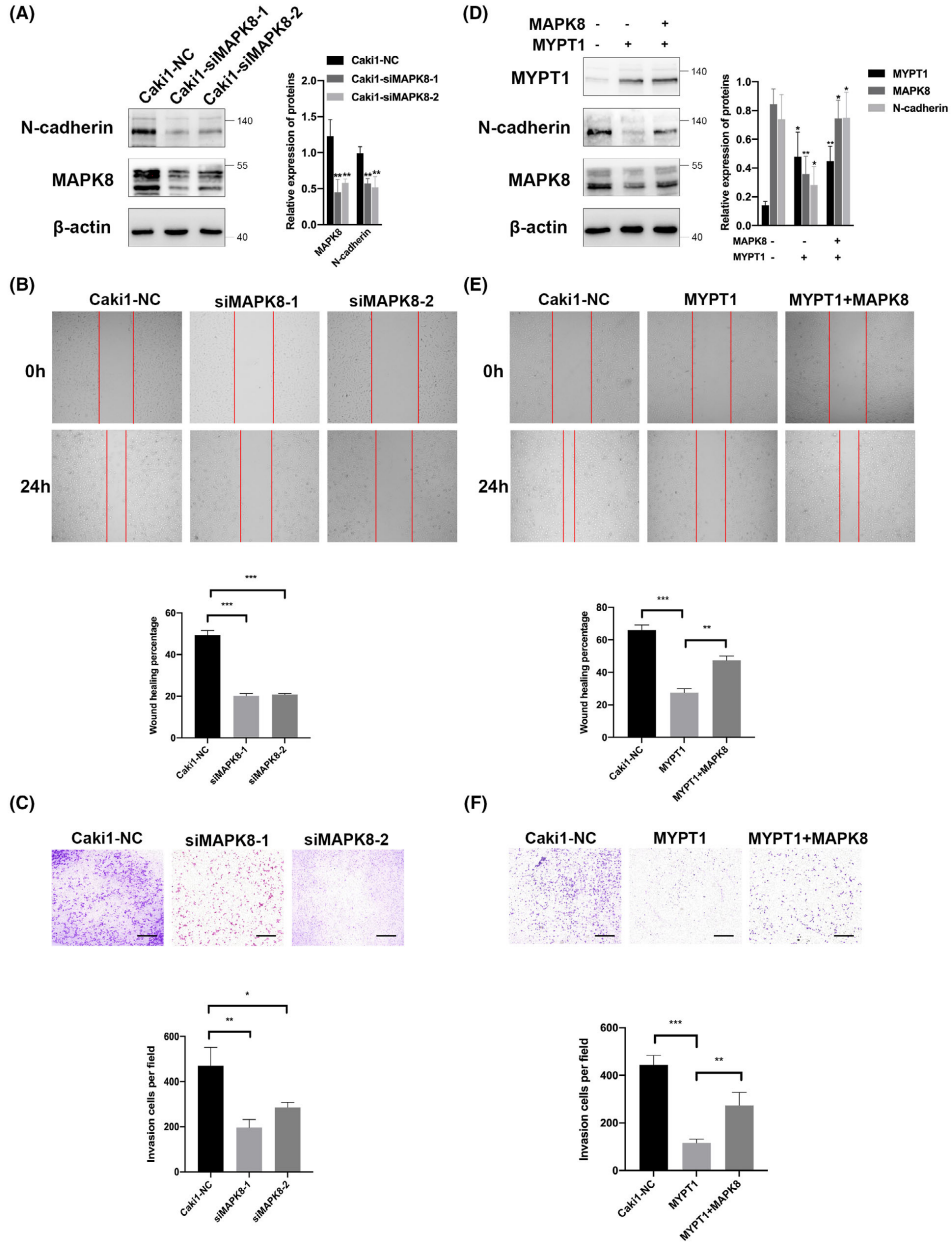
MYPT1 suppressed MAPK8/N‐cadherin expression to influence the migration and invasion of ccRCC cells. (A, D) Western blotting of the indicated proteins in ccRCC cells. The bar plots show the relative band intensities (mean ± SEM) of three independent experiments. (B, E) Cell migration as determined by wound healing assay. Red lines denote the margins of the wound. (C, F) Cell invasion as determined by Transwell assay. Matrigel was added to the upper chamber. Scale bars: 200 μm. Statistical analysis was performed with a two‐tailed unpaired Student's *t* test (*P < 0.05, **P < 0.01, ***P < 0.001). ccRCC, renal clear cell carcinoma; MAPK, mitogen‐activated protein kinase; MYPT1, myosin phosphatase target subunit 1.

MAPK8, which is also termed c‐JUN N‐terminal kinase (JNK), is a MAPK family member. Recent investigations have documented that MAPK8 is activated in cancer progression [15]. By reviewing the literature of the above 17 genes in detail, we found that MAPK8 was required for EMT cell migration and metastasis [16]. Therefore, we speculated that MAPK8 may be involved in the metastasis suppression effect of MYPT1 (Fig. 4E). To determine the role of MAPK8 in ccRCC, we silenced MAPK8 expression in Caki1 cells to explore its influence on ccRCC migration and invasion. As illustrated in Fig. 5A, the knockdown of MAPK8 expression in Caki1 cells resulted in decreased expression of N‐cadherin. In addition, silencing MAPK8 expression reduced cancer cell migration and invasion abilities in ccRCC (*P* < 0.05, Fig. 5B,C). Furthermore, western blotting showed that overexpression of MYPT1 reduced the protein expression of MAPK8, which was consistent with the RNA sequencing data (Fig. 5D). Therefore, we hypothesized that MYPT1 may inhibit N‐cadherin expression, which influences the cancer cell metastasis, by inhibiting the expression of MAPK8. To verify this, we rescued the expression of MAPK8 in Caki1 cells overexpressing MYPT1. Rescue of MAPK8 expression yielded a remarkably strong N‐cadherin signal (Fig. 5D). Additionally, restoration of MAPK8 expression in Caki1‐MYPT1 cells restored their migration and invasion abilities (*P* < 0.05, Fig. 5E,F).

## Discussion

The majority of kidney cancer‐related deaths are linked to ccRCC metastasis [17]. Therefore, determining the molecular mechanisms underlying the metastasis of ccRCC may uncover novel targets for the treatment of this disease. Herein, we illustrated that MYPT1 is downregulated and linked to OS in ccRCC patients. Further studies showed that overexpression of MYPT1 suppressed the migration and invasion of ccRCC cells *in vivo* and *in vitro*. Hence, further investigation into the mechanism is of great value for ccRCC treatment.

MYPT1 belongs to the myosin phosphatase targeting proteins (MYPT) protein family and is not a regulatory but a modulatory subunit of protein phosphatase 1 (PP1). Some research has shown that MYPT1 is recognized as a crucial protein in the smooth muscle myosin phosphorylation module [18, 19] and affects smooth muscle contraction. Emily Joo et al. [7] illustrated that MYPT1 modulates contractility coupled with microtubule acetylation to regulate integrin adhesions and matrix assembly, which helps to regulate the migration rate and epithelial branching morphogenesis. The above data clearly show that MYPT1 plays a role in the migration of cells. In addition, a role in cancer has been described for MYPT1, since MYPT1 is suppressed by miR‐30d to enhance migration and invasion in prostate cancer cells [12]. Adrienn Sipos et al. [20] identified the protein arginine methyltransferase 5 (PRMT5) enzyme of the methylosome complex as a MYPT1‐binding protein uncovering the nuclear MYPT1 interactome of hepatocellular carcinoma cells. These results suggest that MYPT1 may play a crucial role in tumour metastasis. Yang et al. [21] illustrated that the change in MYPT1 phosphorylation was one of the molecular mechanisms through which Rho‐kinase repressor suppresses the proliferation and metastasis of small lung cancer cells. Moreover, we explored and found that MYPT1 expression is downregulated in human renal clear cell carcinoma, and its overexpression in ccRCC cells and xenograft models inhibits metastasis, suggesting its suppressive role in ccRCC metastasis.

EMT is a process in which epithelial cells acquire mesenchymal features. In cancer, EMT is associated with tumour initiation, invasion, metastasis, and resistance to therapy [22]. Epithelial cancer cells, especially renal tubular epithelial cells that arise during embryogenesis by mesenchymal to epithelial transition (MET), are inclined to undergo EMT [23]. Therefore, EMT is thought to be an important event during malignant tumour progression and metastasis [24]. After we have established that MYPT1 overexpression can inhibit the metastasis of ccRCC, we examined the expression of major EMT effectors, including E‐cadherin, N‐cadherin and Vimentin. The results showed that MYPT1 overexpression could significantly inhibit the expression of N‐cadherin but not E‐cadherin or Vimentin, which led us to speculate that MYPT1 suppresses tumour metastasis by inhibiting N‐cadherin.

To fully elucidate the biological function of MYPT1 in ccRCC, we utilized an RNA‐sequencing strategy and performed bioinformatic analysis on MYPT1‐overexpressing cells. The results showed that the MAPK pathway may play significant roles in the anti‐metastatic function of MYPT1. The MAPK signalling cascade is a remarkable cellular cascade triggered in response to DNA damage, and it was also proven to be linked to tumour progression with regard to cell survival, migration and autophagy [25]. In this cascade, mitogen‐activated protein kinase 8 (MAPK8) is phosphorylated and activated and in turn phosphorylates a number of transcription factors, primarily components of AP‐1, such as JUN, JDP2 and ATF2, and thus regulates AP‐1 transcriptional activity [26]. MAPK8 was also found to be activated by tumour necrosis factor alpha (TNF‐alpha) and required for TNF‐alpha‐induced apoptosis [27]. This kinase and its target transcription factor JUN have also been implicated in EMT, which is thought to be related to cancer migration [28, 29]. In line with these observations, MAPK8 phosphorylated paxillin, a focal adhesion adaptor required for the formation of focal adhesion plaques and for efficient cell migration [30]. These data are consistent with our findings that MYPT1 can inhibit N‐cadherin expression and prevent metastasis formation by repressing MAPK8 protein expression.

However, there are still several limitations to this study. The specific mechanism by which MYPT1 affects MAPK8 is unclear. We speculate that this may be related to the function of MYPT1 in regulating myosin phosphatase activity by targeting the holoenzyme to its substrates [31]. PP1c catalytic subunits, including PPP1CA and PPP1CB, are required for the catalytic activity of the myosin phosphatase, while phosphorylation of MYPT1 at Thr696 and Thr853 results in myosin phosphatase inhibition and cytoskeletal reorganization [32, 33]. Therefore, we examined the expression of phospho‐MYPT1 (Thr696 and Thr853), PPP1CA and PPP1CB in MYPT1‐overexpressing and MYPT1‐knockdown cells. The results showed that overexpression or knockdown of MYPT1 positively altered its phosphorylation levels at the Thr853 and Thr696 sites but did not alter the expression of PPP1CA and PPP1CB (Fig. S2). Further in‐depth experimental studies should be conducted to investigate the link between MYPT1 phosphorylation and MAPK8 expression.

## Conclusions

Our findings illustrate that MYPT1 may suppress metastasis via the MAPK8/N‐cadherin pathway and reveal a novel mechanism underlying ccRCC progression. Since anti‐metastasis therapy has been a crucial strategy for the treatment of human cancers, MYPT1 may be a potential drug candidate in anticancer therapy.

## Conflict of interest

The authors declare no conflict of interest.

## Author contributions

QX, RL, ZZ, and YF acquired the data. RL, YF, YH, and GX analysed and interpreted the data. RL and YL contributed MYPT1 constructs. QX, YL, and WZ drafted the manuscript. WS, YL, and WZ handled the funding and supervision. The authors read and approved the final manuscript.

## Supporting information


**Fig. S1.** MYPT1 expression in different renal cell lines.
**Fig. S2.** Overexpression or knockdown of MYPT1 altered its phosphorylation levels at the Thr853 and Thr696 sites but did not alter the expression of PPP1CA and PPP1CB.

## Data Availability

The data that support the findings of this study are available.
