# Supplementary material for: MYPT1 inhibits the metastasis of renal clear cell carcinoma via the MAPK8/N‐cadherin pathway
Source: FEBS Open Bio. 2022 Sep 27;12(11):2083–95. doi: 10.1002/2211-5463.13487 (PMC9623519; doi:10.1002/2211-5463.13487)
Supplement: Supplementary file 1 — Fig. S1. MYPT1 expression in different renal cell lines. Fig. S2. Overexpression or knockdown of MYPT1 altered its phosphorylation levels at the Thr853 and Thr696 sites but did not alter the expression of PPP1CA and PPP1CB. [file FEB4-12-2083-s001.docx]

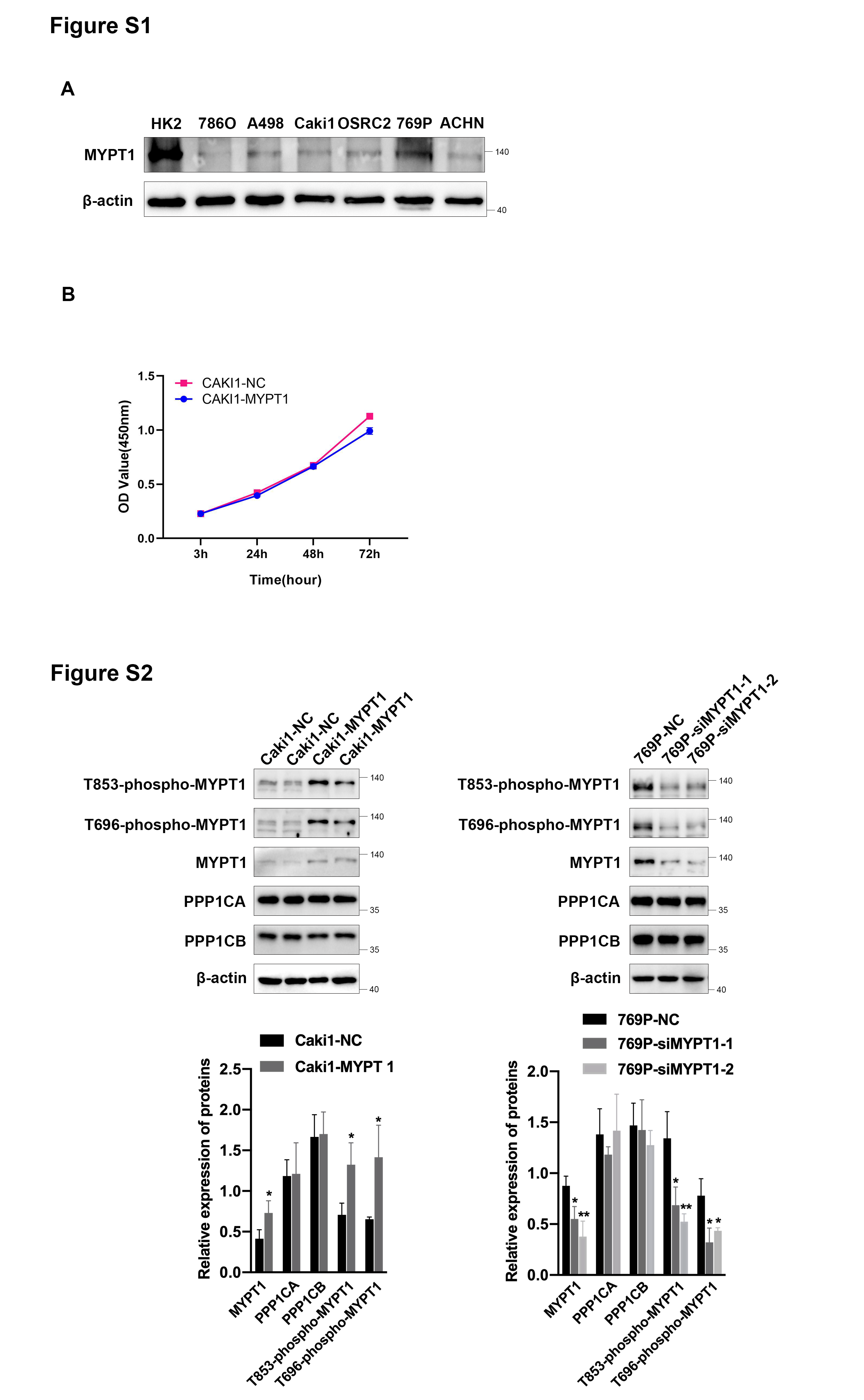


Figure S1. (A) MYPT1 expression was analyzed by Western blotting in different renal cell lines. (B) ﻿Cell proliferation was determined by ﻿cell counting kit.

Figure S2. Proteins were analyzed by Western blotting using the indicated antibodies. ﻿The bar plots show the relative band intensities (mean ± SEM) of three independent experiments. Statistical analysis was performed with a two-tailed unpaired Student’s t test (*P < 0.05, **P < 0.01).
